# Hydrogel Beads Loaded with Glucosinolate-Rich *Brassicaceae* Extract as a Controlled-Release Alternative to Biofumigation

**DOI:** 10.3390/molecules30183660

**Published:** 2025-09-09

**Authors:** Michele Baglioni, Ilaria Clemente, Raffaello Nardin, Flavia Bisozzi, Sara Costantini, Giacomo Fattori, Gabriella Tamasi, Claudio Rossi

**Affiliations:** 1 Department of Biotechnology, Chemistry and Pharmacy, University of Siena, Via Aldo Moro 2, 53100 Siena, Italy; ilaria.clemente2@unisi.it (I.C.); raffaello.nardin2@unisi.it (R.N.); flavia.bisozzi@student.unisi.it (F.B.); sara.costantini@student.unisi.it (S.C.); giacomo.fattori@student.unisi.it (G.F.); gabriella.tamasi@unisi.it (G.T.); claudio.rossi@unisi.it (C.R.); 2 Centre for Colloid and Surface Science (CSGI), University of Florence, Via Della Lastruccia 3, 50019 Sesto Fiorentino, FI, Italy

**Keywords:** alginate, carboxymethyl cellulose, glucoraphanin, agricultural soil, circular economy

## Abstract

Biofumigation was originally proposed as an alternative to toxic fumigants for the treatment of agricultural soils, owing to the biocidal effect of isothiocyanates (ITCs) released by some plant species like *Brassicaceae*. However, biofumigation also presents limitations; thus, an advanced and viable alternative could be the use of controlled-release systems such as gelled polymer networks. In the present work, we explore the use of biocompatible hydrogels based on sodium alginate (ALG) and sodium carboxymethylcellulose (CMC), conveniently loaded with a Brassicaceae extract for this purpose. The extract was characterized by means of HPLC-MS, showing its high glucosinolate content, especially glucoraphanin, a secondary metabolite produced by several species of this family. The physicochemical properties of the synthesized gels were investigated by means of differential scanning calorimetry (DSC), rheometry, and scanning electron microscopy (SEM), both in the presence and absence of the loaded extract. Loading and release kinetics (in water) were studied by means of HPLC-DAD, and the Weibull model was employed to interpret the results. It was found that both hydrogels can effectively confine the Brassicaceae extract’s active principle, slowly releasing it in an aqueous environment. Both systems possess excellent properties for real applications, with the CMC-based hydrogels being slightly preferable over the ALG ones due to their higher encapsulation efficiency, mechanical properties, and overall features. These systems are promising tools for combating harmful microorganisms due to the biocidal properties of glucosinolates, but their potential goes beyond their use in agriculture, as they could be applied as antifouling or antimicrobial agents in cultural heritage cleaning or other fields.

## 1. Introduction

The increasing global demand for higher crop yields has led to the widespread use of chemical treatments in agriculture. In this context, synthetic pesticides represent a particular concern due to their adverse effects on human health and the environment [1,2,3,4,5,6]. Thus, the challenge of balancing the control of effective soilborne pathogens with sustainability and safety has become a major focus in modern agrifood research.

Soil fumigants such as metam sodium (commercially known as Vapam), metam potassium (Tamifum)—chemically, the sodium or potassium salts of dithiocarbamate, respectively—, and dazomet (3,5-dimethyl-1,3,5-thiadiazinane-2-thione) have been widely used against a range of soilborne organisms. Several of these are potentially harmful to crops, including insects, fungi, bacteria, and nematodes. These chemicals were introduced as less harmful alternatives to methyl bromide. CH_3_Br is a highly effective fumigant whose use was severely limited by the 1987 Montreal Protocol due to its toxicity and its role in ozone layer depletion [7,8,9,10,11].

A well-known and viable alternative to chemical fumigants is biofumigation [12,13,14,15,16,17]. In this method, fresh plants (mostly *Brassicaceae*) are cultivated, mown, and mixed into agricultural soils due to their biocidal properties against soilborne pathogens. *Brassicaceae* are characterized by high glucosinolate content, i.e., secondary metabolites produced by plants for defensive purposes. Glucosinolates can be hydrolyzed to isothiocyanates (ITCs) by the enzyme myrosinase.

In plant cells, glucosinolates and myrosinase are located in different compartments, so enzymatic hydrolysis can only be induced by mechanical tissue damage (grinding, mowing, etc.) [18]. Myrosinase-catalyzed hydrolysis of glucosinolates initially involves the cleavage of the thioglucoside bond, thus forming D-glucose and thiohydroximate-O-sulfonate. The latter is unstable and spontaneously forms a wide range of products, such as thiocyanates, nitriles, and, as mentioned, ITCs. These are responsible for the defensive mechanisms of plants against soilborne phytopathogens, fungi, and insects.

The same principle is, in fact, used in biofumigation for the treatment of agricultural soils, owing to the biocidal effect of ITCs and the other chemicals derived from glucosinolates. The effectiveness of ITCs in inhibiting the growth of several microorganisms, such as the fungi *Rhizoctonia solani*, *Sclerotinia minor*, *Sclerotinia sclerotiorum*, *Alternaria brassicicola*, and *Fusarium oxysporum*, as well as other plant pathogens, has been extensively reported in the literature [19,20,21,22]. ITCs owe their biocidal properties to the strong electrophilicity of the isothiocyanate group’s carbon, which is highly reactive with thiols, amines, and alcohols [21], thus readily interacting with SH groups in proteins, causing mutations in vivo that can interfere with the regular biochemical processes of cells [16,23].

However, biofumigation also has some limitations: (i) it is a time-consuming practice; (ii) the amount of glucosinolate/myrosinase delivered to the soil is hardly controllable [24]; and (iii) it is a relatively ineffective agricultural practice [25]. Some improvements have been developed over time, such as the introduction of powdered *Brassicaceae* “flours” or dry pellets [24], but research into alternative approaches for soil treatment has progressed, finding more reliable, more effective, and safer solutions.

A notable contribution to the search for more effective and high-performing alternatives to biofumigation is controlled-release (CR) systems for the delivery of several chemicals to agricultural soil. Several different strategies have been proposed to develop CR systems for different classes of active chemicals [26,27,28,29,30,31,32,33], but one of the main and most effective is hydrogels based on biodegradable polymers [34,35,36,37].

Several polymers (e.g., chitosan, alginate, pectin, or modified celluloses) have been proposed to synthesize gelled networks that can be used as carriers for active chemicals, such as pesticides (e.g., diuron, carbofuran, atrazine, isoproturon, imidacloprid, cyromazine, chloridazon, and metribuzin [38]); fertilizers (e.g., nitrogen, phosphoros, and potassium—NPK [29]); plant growth inhibitors or retardants (e.g., paclobutrazol, flurprimidol, and uniconazole [26]); and other active substances [27,30].

However, it is evident that the state of the art in the field is mainly limited to systems for the CR of synthetic pesticides. Even if this represents an improvement over the uncontrolled use of free pesticides, fertilizers, and other chemicals, a further step toward sustainability needs to be taken.

Borrowing the basic ideas and concepts behind biofumigation and aiming at a zero-waste circular economy concept, byproducts from agrifood supply chains can represent a source of extremely interesting bioactive compounds. Several (nano)carrier systems have been proposed for CR, where either the vector matrix or the bioactive compounds (or both) were derived from byproducts of agrifood production [39,40,41,42,43,44]. Food waste from *Brassicaceae* crops is a rich source of glucosinolates, which can be exploited for eco-friendly antimicrobial soil treatment as a viable alternative to synthetic fumigants. By encapsulating bioactive plant-derived compounds into hydrogel matrices, it is possible to develop innovative soil treatment methods that combine the benefits of biofumigation with CR principles. Hydrogel beads loaded with ITC-based microemulsions have recently been proposed as an enhanced and innovative alternative to biofumigation [45].

This study proposes an approach that represents a step forward from that idea. Hydrogels based on alginate (ALG) and carboxymethylcellulose (CMC) were synthesized and loaded with a commercial *Brassica oleracea* var. *Italica* (broccoli) extract for the advanced biofumigation of agricultural soils. The extract is rich in glucoraphanin (GRF), as shown through the HPLC-MS analyses reported in the present work. Glucoraphanin is a glucosinolate precursor to sulforaphane, a well-known ITC with antimicrobial and pesticidal properties, making it an ideal candidate for soil treatment applications. The choice to load ALG and CMC hydrogels with a *Brassicaceae* extract rich in glucosinolates—particularly glucoraphanin—was based on the premise that myrosinase is produced by soil microbiota and is therefore naturally present in agricultural soils [16]. Therefore, for the purposes of this study, glucoraphanin (and other glucosinolates) can be effectively considered the precursors of the actual biofumigant agents, i.e., ITCs such as sulforaphane or similar.

On the other hand, the choice of ALG and CMC was based on the vast literature on their use as cheap, easily synthesized, and reliable hydrogel carriers for drug delivery in several applicative contexts. Several other biocompatible and biodegradable polymers were initially considered, including chitosan and pectin, but we eventually focused on the most promising systems, i.e., ALG- and CMC-based hydrogel beads.

The synthesized hydrogels were characterized by means of attenuated total reflectance—Fourier transform infrared spectroscopy (ATR-FTIR), differential scanning calorimetry (DSC, for the determination of the free water index), scanning electron microscopy (SEM), and rheological analysis to determine their structural and physicochemical properties in the presence or absence of the encapsulated extract. Furthermore, the loading and release kinetics of the active compound in aqueous media were studied to assess their potential as a sustainable alternative for soil treatment. By combining hydrogel technology with plant-derived biocidal compounds, this study aims to contribute to the development of effective and environmentally responsible strategies for agricultural soil management, reducing reliance on synthetic pesticides and advancing sustainable agricultural practices.

## 2. Results and Discussion

### 2.1. Brassica Oleracea Extract Characterization

**Figure 1:**
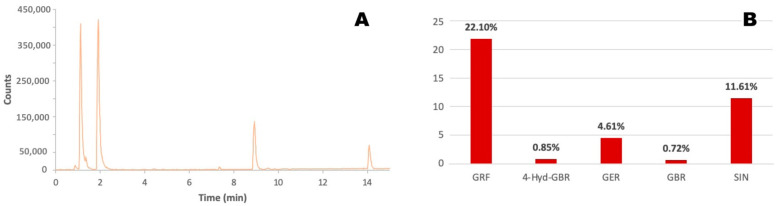
(**A**) Chromatogram obtained from the HPLC-DAD analysis for the characterization of the *Brassica oleracea* extract. (**B**) Relative amounts of the most abundant compounds identified in the *Brassica oleracea* extract. GRF: glucoraphanin, 22.10 ± 0.99%; 4-Hyd-GBR: 4-hydroxyglucobrassicin, 0.85 ± 0.04%; GER: glucoerucin, 4.61 ± 0.21%; GBR: glucobrassicin, 0.72 ± 0.03%; SIN: sinapine, 11.61 ± 0.52%.

First, the *Brassica oleracea* extract was characterized by means of HPLC-MS and HPLC-DAD analyses, as described in Section 3.3, to identify and quantify the main chemicals present. The chromatogram obtained from the chromatographic analysis is shown in Figure 1A, where peaks corresponding to the most abundant compounds present in the extract are clearly visible. The detected analytes were fragmented using tandem mass spectrometry (MSⁿ), with He as the collision gas inside the ion trap, in negative ion mode, according to the method described in Section 3.3. The use of fragmentation patterns enabled the identification of the compounds listed in Table 1.

Several diagnostic ions for glucosinolates have been reported in the literature, e.g., those related to the loss of the HSO_3_^−^ ion (*m*/*z* 96 or 97) and the neutral loss of the glucose moiety (*m*/*z* 162) [46]. Glucoraphanin was clearly visible in the chromatogram at a retention time of 1.91 min, and the deprotonated molecule [M − H]^−^ was identified through comparison with literature data [46]. Besides glucoraphanin, other glucosinolates were present in the extract in significant amounts, such as 4-hydroxyglucobrassicin (an indolic glucosinolate derived from glucobrassicin), glucoerucin, and glucobrassicin, identified through their characteristic MS2 fragments [46], as reported in Table 1.

The elution peak visible at 14.09 min in the chromatogram in Figure 1A was attributed to sinapine, an alkaloid derived from sinapic acid and commonly found in seeds of certain *Brassicaceae* plants [47]. Other minor compounds were also detected in the extract, but they were not identified.

The identified glucosinolates and sinapine were then quantified by means of HPLC-DAD analyses, and the results are reported in Figure 1B, showing a total glucosinolate content of 28.28 ± 1.27%. It is worth noting that the relatively high content (11.61 ± 0.52%) of sinapine can also contribute to the antimicrobial activity of the extract due to the demonstrated antimicrobial properties of this alkaloid [48].

### 2.2. Physico-Chemical Characterization of ALG and CMC Hydrogels

The hydrogels based on ALG and CMC polymers were then synthesized, exploring several different extract concentrations, i.e., 0%, 1%, 2.5%, and 5% (with respect to the initial polymeric solution). The equilibrium water content (EWC), calculated as described in Section 3.4, was found to be 93 ± 1% for all gels, both unloaded and loaded with the extract.

The FWI of the developed systems was then obtained through DSC analysis (see Section 3.5), and the results determined for all samples are reported in Table 2.

As shown in Table 2, a decrease in the FWI was observed in the CMC hydrogels with increasing extract concentration. Conversely, the FWI tended to increase for the alginate hydrogels loaded with higher amounts of extract. This effect was particularly pronounced in systems with higher concentrations (2.5% and 5%), where the FWI difference was more significant. In contrast, the difference between pure alginate and alginate loaded with 1% extract was smaller. This behavior was likely due to a complex and synergistic effect that took into account the chemical nature of the two polymers; their interactions with Ca^2+^ and Fe^3+^ cations, respectively; and their interactions with the variety of compounds included in the extract. It is indeed known that glucosinolates (particularly glucoraphanin and glucobrassicin) are prone to complexation with iron ions [49,50,51], while less evidence is present in the literature with respect to interactions with calcium ions. More specifically, Fe^2+^ ions are responsible for inducing non-enzymatic and thermal degradation of glucosinolates, while no such effects are observed for Fe^3+^, which can still form stable complexes. Thus, it can be inferred that the glucosinolate/Fe^3+^ interactions were ultimately responsible for some water structuring in the proximity of the formed complexes, and this was observed as a consistent reduction in the FWI for the CMC/Fe^3+^ hydrogel systems. On the other hand, the significant increase in the FWI for the ALG-based systems (especially at the 2.5% and 5% extract concentration) can be attributed to better interactions between alginate chains and the extract’s compounds (compared to carboxymethyl cellulose/glucosinolate interaction), which replaced water molecules inside the gel’s walls. This resulted in a weakening of the polymer network structure, which was further confirmed by rheological analyses of the hydrogels.

**Figure 2:**
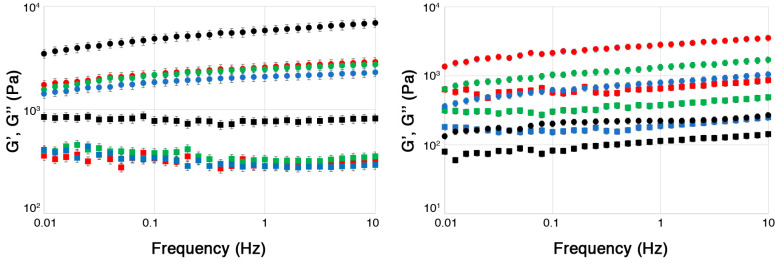
Frequency sweep graphs showing G’ (circles) and G’’ (squares) moduli for all hydrogels: (**Left**) CMC hydrogel series; (**Right**) ALG hydrogel series. Black: unloaded gels, initial extract concentration = 0%; red: initial extract concentration = 1%; green: initial extract concentration = 2.5%; blue: initial extract concentration = 5%. The measurements were carried out by selecting an amplitude in the linear viscoelastic region, identified through amplitude sweep analyses.

Figure 2 shows the frequency sweep graphs reporting the storage (G’) and loss (G’’) moduli measured for all hydrogels.

All systems exhibited a predominantly elastic behavior (characteristic of solid-like viscoelastic systems), as G’ was larger than G’’ across the entire frequency range explored, and the CMC-based systems showed higher mechanical properties overall, as evidenced by the comparison between the absolute value of the two moduli for the systems based on the two different polymers. This was likely due to the crosslinking effect of Fe^3+^ ions, which are known to form stiffer and mechanically more resistant gels than Ca^2+^ [52].

Most interestingly, it was observed that for the ALG hydrogels, after an initial and abrupt increase between ALG and ALG-1, both G’ and G’’ decreased for higher extract concentrations. This suggests that the hydrogel structure, even if initially strengthened by the addition of the extract, was weakened by increasing the amount of glucosinolates in the hydrogel. These results are consistent with the DSC findings, which suggest that the increase in the FWI for the ALG-based systems was likely due to the competition between extract and water molecules when interacting with the alginate chains, weakening the hydrogel structure.

Conversely, in the case of the CMC-based systems, only the unloaded hydrogel had significantly higher moduli compared to the extract-loaded systems, which all showed similar mechanical properties that evidence a generally weaker structure. These findings also reinforce the idea that the FWI decrease observed for these systems is indeed related to the formation of glucosinolate/Fe^3+^ hydrated complexes, rather than to an increased hydration of carboxymethyl cellulose chains, as proposed above when discussing the DSC data.

SEM micrographs of the ALG and CMC-based hydrogels, both loaded and unloaded, were then taken to collect information on the micromorphology of these systems.

**Figure 3:**
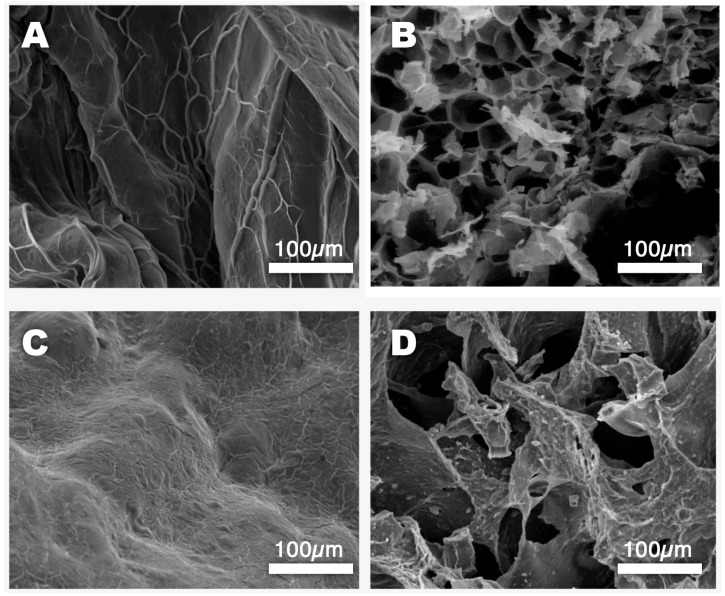
SEM micrographs taken on the freeze-dried hydrogels. (**A**) Surface of an ALG-0 hydrogel bead; (**B**) Cross-section of an ALG-5 hydrogel bead; (**C**) Surface of a CMC-0 hydrogel bead; (**D**) Cross-section of a CMC-5 hydrogel bead—some extract clumps are visible on the gel walls.

Figure 3A shows the surface of an unloaded alginate bead (ALG-0). Some surface roughness is visible as a result of a slight deformation that occurred during freeze-drying, even though the process was designed to minimize material stress. The surface of the unloaded CMC-0 hydrogel bead is shown in Figure 3C. This system seems to have better withstood the dehydration process compared to the ALG one, in agreement with the rheology analyses that proved that the carboxymethyl cellulose-based systems had higher mechanical resistance than the alginate-based ones. Figure 3B,D show a cross-section of both kinds of hydrogel beads, allowing a look at their inner structures. Interestingly, both gels have large pores, which is a well-established feature of alginate/Ca^2+^ hydrogels, according to the existing literature on these systems [53]. That being said, the structures of the two gels are quite different, with CMC-0 characterized by a more inhomogeneous network of thicker gel’s walls intercalated by larger pores. This seemingly more robust structure was reflected in the mechanical properties of the hydrogel, as discussed above.

The encapsulation efficiency (EE%) was then measured, as described in Section 3.8.1, as it is a crucial parameter for the characterization of CR systems. In this case, it represents the amount of the active principle retained inside the hydrogels with respect to the amount initially mixed with the polymer solution. Knowing this parameter is necessary for calculating the subsequent release efficiency (RE%) of the hydrogel beads and provides insight into the nature of the interactions between the polymer network and the extract’s compounds. As mentioned above, in the present case, glucoraphanin was quantified and taken as a marker to measure the loading and release profiles, being the main glucosinolate present in the *Brassica oleracea* extract.

**Figure 4:**
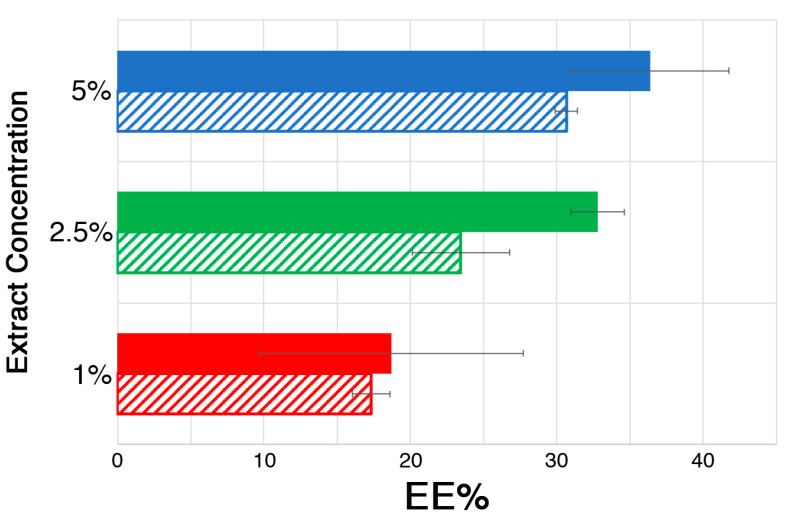
EE% for the ALG (hatched bars) and CMC (solid bars) hydrogels loaded with different extract concentrations.

Figure 4 compares the EE% values of the ALG and CMC hydrogels for different initial extract concentrations (1%, 2.5%, and 5%). Looking at the histograms, two main features appear to be evident, even though the experimental errors in some cases are such that some observed differences in average values actually need to be handled cautiously: (i) the EE% for the CMC-based hydrogels was higher than that for the ALG-based systems; (ii) the EE% increased as the extract concentration increased in the initial polymer solution. These two trends need to be discussed separately.

**Figure 5:**
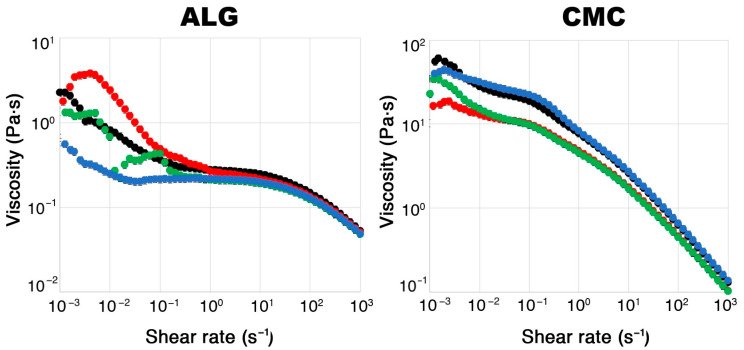
Viscosity of ALG (**Left**) and CMC (**Right**) polymeric solutions (with different extract concentrations) before crosslinking. Black: unloaded gels, initial extract concentration = 0%; red: initial extract concentration = 1%; green: initial extract concentration = 2.5%; blue: initial extract concentration = 5%. Error bars are not clearly visible, as they are smaller than the markers.

The first could suggest that carboxymethyl cellulose has a greater affinity for glucoraphanin (as this study is related to this specific glucosinolate) with respect to alginate. This would result in a lower loss of this compound in the crosslinking solution during the gelation process or during subsequent hydrogel rinsing. However, when looking at the FWI data, as discussed before, this did not seem to be the case. A more convincing hypothesis is the aforementioned likely formation of glucosinolate/Fe^3+^ complexes, which could hinder glucoraphanin migration from inside the gel, together with the contribution of the system’s inner structure, characterized by thick gel walls that may be more efficient at confining the active compound. However, another important factor must be considered, i.e., the average size of the hydrogel beads for the ALG and CMC systems. Figure 5 shows that the CMC solution is much more viscous than the ALG one, as visible in the rheology measurements of polymeric solutions with different extract concentrations.

**Figure 6:**
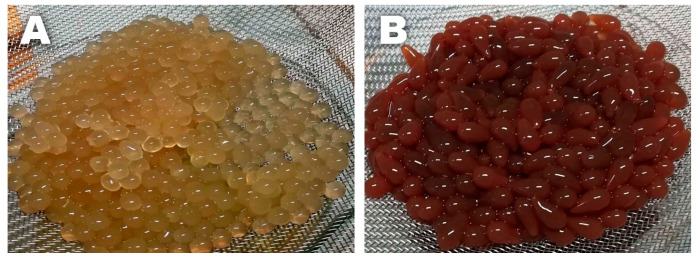
(**A**) ALG-5 hydrogel beads; (**B**) CMC-5 hydrogel beads. The pictures were taken at the same scale, and it can be seen that the CMC beads are larger than the ALG ones. The CMC hydrogels also have a darker color due to the presence of Fe^3+^ ions in the polymer network.

Apart from some not easily explainable behavior (especially for the ALG polymer solutions, possibly due to the complex influence of the extract’s molecules on the rheological properties) at a low shear rate, both polymer solutions (even in the presence of increasing extract concentrations) exhibited a shear-thinning trend in their viscosity, which is typical of non-Newtonian fluids. However, the most noteworthy difference is that the CMC polymer solutions had a viscosity of about one order of magnitude higher than that of the ALG solutions. The difference was so marked that this was also visible to the naked eye. This resulted in the CMC solutions dripping more slowly from the funnel than the ALG ones during the crosslinking process, thus forming larger hydrogel beads on average, with a slightly more elongated shape (see Figure 6). Since the EE% depends on the amount of glucoraphanin lost in the crosslinker solution and the rinsing water as a result of glucosinolate migration from the gel to the outer environment (driven by a concentration gradient), it seems reasonable that it is also related to the surface-to-volume (S/V) ratio of the hydrogel beads. The sizes of the CMC and ALG hydrogel beads were then measured using a digital caliper. The measurements revealed that the bead sizes did not significantly change with the extract concentration for either hydrogel. The ALG beads had a roughly spherical shape, characterized by an S/V_ALG_ of 1.7 ± 0.1 mm^−1^. On the other hand, the CMC beads were approximated as prolate ellipsoids, with an S/V_CMC_ of 1.13 ± 0.04 mm^−1^. Indeed, this means that, for an equal volume of hydrogel, the interface between the gel beads and the outer aqueous environment was smaller for the CMC-based systems, which could be more efficient for retaining glucoraphanin inside the gel matrix.

The second feature observed in the EE% of the investigated systems was related to the fact that the encapsulation efficiency increased with the extract concentration. It is worth noting that the absolute amount of glucoraphanin lost in the crosslinker saline solution was higher for higher extract concentrations; thus, one could have expected a constant EE% over the whole range of concentrations explored. However, this was not the case because, even though the increase in the initial internal glucoraphanin concentration inside the forming gel bead would enlarge the concentration gradient that drives active compound migration, the interfacial area was the same as for lower concentrations, meaning that the process has a self-limiting (quasi) asymptotic trend. For this reason, the amount of glucoraphanin retained inside the hydrogel (i.e., the EE%) increased with increasing extract concentration.

**Figure 7:**
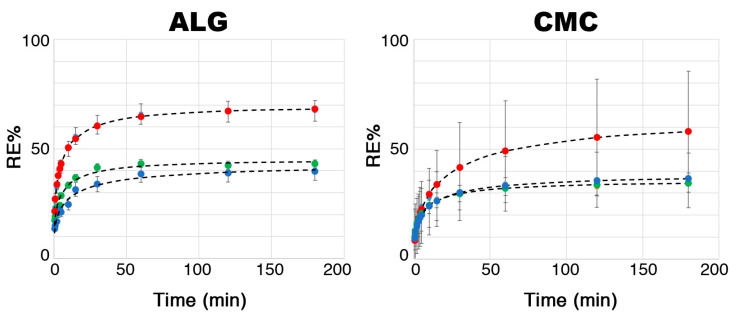
Release kinetics profiles of glucoraphanin for all loaded systems investigated. (**Left**) ALG hydrogels; (**Right**) CMC hydrogels. Red: initial extract concentration = 1%; green: initial extract concentration = 2.5%; blue: initial extract concentration = 5%. Dashed black lines represent the best-fitting curve for each system, computed using the Weibull model.

The release efficiency (RE%) is another key parameter for characterizing hydrogels to be used as CR media, as it indicates their ability to release the active compound—a crucial step in the treatment of agricultural soils. Before studying the behavior of the loaded hydrogel beads in simulated or actual soils, their release profiles were investigated in water to compute a model system. In fact, since glucoraphanin is a water-soluble compound, it can be hypothesized that its release in soil also occurs primarily through soil moisture. By knowing the initial amount of glucoraphanin confined in the investigated systems (by exploiting the EE% values previously measured, as discussed above) and measuring the glucoraphanin (taken again as the reference glucosinolate, representative of all active compounds in the extract) released by the hydrogel beads immersed in a known amount of water (see Section 3.8.2) via HPLC-DAD, it was possible to plot the release profiles, as shown in Figure 7.

For both the ALG- and CMC-based hydrogels, glucoraphanin release in water showed seemingly asymptotic behavior. In fact, the RE% was found to decrease as the extract concentration increased, showing an opposite trend to what was observed for the EE%, where higher values were obtained for hydrogels initially loaded with 5% extract. These results were expected because, in this case, a high EE% implies that the hydrogel better retained glucoraphanin. As further confirmation, it was observed that the RE% of the CMC-based systems was generally lower than that of the ALG-based ones. Furthermore, for both the ALG and CMC systems, it was noted that the RE% leveled out at around 40% of the initial content of the active compound as the extract concentration increased. It is worth noting, however, that even though the CMC-1 and ALG-1 systems had higher maximum RE%, the absolute amount of glucoraphanin released for CMC-5 and ALG-5 was found to be 5–6 times higher after 3 h of release in water (see the last column of Table 3).

Many suitable models can be used to fit the release kinetics of porous matrices, such as hydrogels or mesoporous particles, e.g., those proposed by Higuchi et al. [54] or Peppas and coworkers [55,56,57]. Such models represent short-time approximations of kinetic curves related to diffusion processes, which can conveniently account for approx. 60% of the whole curve [58]. In this case, similarly to what has been done before on similar systems [45,59], the Weibull model was used. This is a modified version of first-order kinetics models, and it is described by Equation (1):

(1) $$C_{t}=C_{\infty }(1-e^{-{at}^{b}})$$

where *C_t_* is the experimental concentration measured in the water in which the hydrogels were immersed as a function of time; $C_{\infty }$ is the maximum concentration reached at infinite time (an asymptotic value); *t* is the time the hydrogels were immersed in water; *a* is a dimensionless empirical constant; and *b* is another dimensionless constant, which has been shown to have a linear correlation with the Peppas exponent, *n*, as reported in the following Equation (2):

(2) $$\frac{C_{t}}{C_{\infty }}=kt^{n}$$

It has been demonstrated that if *b* < 0.75, the process is governed by Fickian diffusion; if 0.75 < *b* < 1, a combination of Fickian diffusion and Case II diffusion takes place, while for *b* > 1, a complex diffusion mechanism occurs [59]. In the present case, Equation (1) was normalized by the total concentration of glucoraphanin initially present in each hydrogel system (*C_TOT_*), and this gave Equation (3), which was then used to fit the experimental data and to obtain the ER%:

(3) $$\frac{C_{t}}{C_{TOT}}=\frac{C_{\infty }}{C_{TOT}}\left(1-e^{-{at}^{b}}\right)=ER\%=K\left(1-e^{-{at}^{b}}\right)$$

Table 3 shows the values obtained for the fitting parameters (*K*, *a*, *b*) for all investigated systems. The best-fitting curves are shown together with the experimental data in Figure 7. The Weibull model provided an excellent fit for all six systems. Since the values of the dimensionless constant *b* were all below 0.75, the release of glucoraphanin in water followed Fickian diffusion kinetics.

The parameter *a* is a measurement of how fast the release is at the early stage, and it can be appreciated here that the ALG-based hydrogel beads are less retentive with respect to glucoraphanin, which is in good agreement with previously reported findings. The release process in water, although it only roughly approximates the real case, provides evidence that glucosinolate delivery is slow and gradual. This would increase control over the fumigation potential in agricultural soils. Finally, it is worth noting that, in a real soil treatment, hydrogel beads would act well beyond 3 h, until complete disruption of the gel network and subsequent release of the active compounds, ensuring a durable effect over time.

## 3. Materials and Methods

### 3.1. Chemicals

CaCl_2_·2H_2_O (purity ≥ 99%) and FeCl_3_·6H_2_O (purity ≥ 99%) were purchased from Sigma Aldrich/Merck (Darmstadt, Germany) and used without further purification. Food-grade sodium alginate and sodium carboxymethylcellulose polymers were purchased from Sigma Aldrich and Reoper GmbH (Hamburg, Germany), respectively. The *Brassica oleracea* extract was purchased from Shanghai Qionghui Industrial Co. (Shanghai, China). Glucoraphanin potassium salt (purity ≥ 98%; Extrasynthese, Lyon, France) and sinapine chloride (purity ≥ 98%; Extrasynthese, Lyon, France) were used as analytical standards for the extract’s characterization performed via HPLC-MS. MilliQ Ultrapure (Merck Millipore, Darmstadt, Germany) water was used.

### 3.2. Hydrogel Synthesis

Crosslinked hydrogel beads were prepared similarly to what was described elsewhere [60], starting with 1% *m*/*v* aqueous ALG and CMC solutions. These polymer solutions were added dropwise into saline solutions of 0.3 M CaCl_2_ (for alginate) and 0.3 M FeCl_3_ (for CMC), respectively, at room temperature. These concentrations were preliminarily optimized to obtain gel beads that were mechanically resistant to handling. The promptly formed hydrogel beads (5–8 mm diameter) were then magnetically stirred for 15 min, removed from the crosslinking solutions, washed with distilled water (to remove any unreacted metal ions from the gels’ surface), and stored in polyethylene containers at their equilibrium swelling degree in a slight excess of water. The 15 min stirring time in the crosslinking solution was assessed using preliminary tests to find the optimal compromise between suitable encapsulation efficiency and the hydrogel beads’ mechanical properties. The preparation of the hydrogel beads loaded with the *Brassica oleracea* extract was carried out similarly by alternatively mixing 1%, 2.5%, or 5% *m*/*m* of the extract powder with the two polymer solutions right before bead formation. Samples were named as ALG-0, ALG-1, ALG-2.5, ALG-5, or CMC-0, CMC-1, CMC-2.5, and CMC-5, indicating the polymeric nature of the hydrogel network and the extract concentration.

### 3.3. Brassica Oleracea Extract Characterization

The commercial *Brassica oleracea* extract was first characterized using an HPLC instrument (Thermo Scientific UltiMate 3000, Waltham, MA, USA), operated using the Xcalibur software (Version 4.3, Thermo-Scientific, Waltham, MA, USA) and coupled to a mass spectrometer (Thermo-Scientific LTQ XL, Waltham, MA, USA) equipped with a linear ion trap analyzer, with ESI (electrospray ionization) as the ionization technique. For chromatographic separation, a C18 Polar Phenomenex Kinetex column (150 × 2.1 mm, 2.6 μm, 100 Å) was used in combination with a Phenomenex C18 Polar (2 × 2.1 mm) pre-column, thermostated at 35 ± 1 °C.

The solvents used as the mobile phase were A (H_2_O/formic acid 0.1%, *v*/*v*) and B (acetonitrile/formic acid 0.1%, *v*/*v*), and the following gradient was set up: 0–3 min 0% B (isocratic), 3–20 min 25% B (linear), 20–25 min 50% B (linear), 25–30 min 50% B (isocratic), 30–31 min 0% B (linear), 31–40 min 0% B (isocratic).

Each sample was analyzed in triplicate by injecting 3 μL at a flow rate of 0.4 mL/min. In more detail, an aqueous solution (1% *m*/*v*) of the extract was filtered using a Whatman PTFE syringe filter with a pore size of 0.2 μm and then analyzed. The mass spectra obtained by injecting the eluted analytes into the mass spectrometer were used to identify the main compounds present in the extract. The parameters used for ESI were spray voltage, 3000 V; sheath gas pressure, 35 AU; auxiliary gas pressure, 30 AU; capillary temperature, 350 °C. The analytes in the extract were identified by comparing the MSⁿ spectra, acquired via negative ionization [M − H]^−^, with those reported in the literature.

The quantitative determination of glucosinolate content in the extracts was performed using HPLC coupled with UV spectroscopy (HPLC-DAD) using an RS-3000 Diode Array Detector (Thermo-Scientific) at 230 nm. The gradient used for this chromatographic run was 0–3 min 0% B (isocratic), 3–5 min 70%B (linear), 5–8 min 70% B (isocratic), 8–10 min 0% B (linear), 10–15 min 0% B (isocratic). The glucoraphanin and glucosinolates in the extract (quantified as glucoraphanin equivalents) were quantified using external calibration. The calibration curves (R^2^ > 0.9986 for both linear fittings) for glucoraphanin and sinapine were obtained by injecting standard solutions within the linear concentration range of 0.001–0.1 mM. The LOD and LOQ values were 0.0003 and 0.001 mM, respectively.

### 3.4. Equilibrium Water Content (EWC)

The EWC of the hydrogel beads was measured gravimetrically by completely drying each sample and weighing it before and after. All samples were left to equilibrate beforehand by releasing excess water. The EWC was then calculated using Equation (4) [61]:

(4) $$EWC=\frac{W_{wet}-W_{dry}}{W_{wet}}\times 100$$

where *W*_wet_ is the weight of the swollen hydrogel and *W*_dry_ is the weight of the completely dry hydrogel (i.e., the sole polymeric network).

### 3.5. Differential Scanning Calorimetry (DSC) and Free Water Index (FWI)

DSC measurements were performed to calculate the free water index (FWI) of the gel systems and were carried out on a DSC Q1000 (TA Instruments, New Castle, DE, USA), using sealed aluminum pans under an inert nitrogen atmosphere (nitrogen flow: 50.0 ± 0.5 cm^3^/min). The samples were equilibrated at −60 °C for 8 min, then heated from −60 °C up to 25 °C at 1 °C/min. The calculation of the FWI from the enthalpy of the fusion values (obtained from the integration of the DSC curve peak around 0 °C) was performed according to Equation (5) [62]:

(5) $$FWI=\frac{{\Delta H}_{fus(exp)}}{EWC\cdot {\Delta H}_{fus(theo)}}$$

where ΔH_fus (exp)_ (J/g) is the experimental value of the enthalpy variation relative to the melting of frozen free water and ΔH_fus (theo)_ (333.1 J/g) is the theoretical value of the enthalpy of fusion for bulk water.

### 3.6. Scanning Electron Microscopy (SEM)

Scanning electron micrographs of pristine and loaded ALG and CMC gels were taken using a Quanta 400 SEM apparatus (FEI Company, Hillsboro, OR, USA) operating at a voltage of 20 kV. The hydrogel samples were freeze-dried to allow their investigation in high-vacuum conditions. Subsequently, they were placed onto stubs using double-sided conductive tape and sputter-coated with gold to make them conductive.

### 3.7. Rheometry on Polymer Solutions and Hydrogels

Rheological analyses were performed using a Discovery HR-2 rheometer (TA Instruments, New Castle, DE, USA) on CMC and ALG systems for all extract concentrations explored (0%, 1%, 2.5%, and 5%).

First, the viscosity of the ALG and CMC polymeric solutions (before the crosslinking gelation process) was determined as a function of the shear rate (1/s), using a rotational rheometer with cone–plate geometry.

Then, the determination of the G′ and G″ moduli was carried out on already formed hydrogels in the form of discs instead of beads. To obtain this gel shape (more suitable for rheometry), each system was crosslinked by pouring it into a Petri dish containing the crosslinker solution. Additional solution was added dropwise using a Pasteur pipette to reach an excess of cations, as in the hydrogel synthesis process described in Section 3.2. After 15 min, the crosslinker solutions were removed, and the hydrogels were left to rest for one day and stored, as in the previously analyzed systems. Then, they were washed and analyzed using the plate–plate geometry to avoid inconsistencies in force distribution.

### 3.8. Encapsulation Efficiency and Release Profiles

#### 3.8.1. Encapsulation Efficiency (EE%)

The loading kinetics and the encapsulation efficiency (EE%) were determined focusing on glucoraphanin, the most abundant glucosinolate found in the commercial *Brassica oleracea* extract. The EE% was calculated using the following equation:

(6) $$EE\%=\frac{{GRF}_{sol}-{GRF}_{lost}}{{GRF}_{sol}}\times 100$$

where *GRF_sol_* is the amount of glucoraphanin initially mixed in the polymer solution (its value is simply obtained by multiplying the mass of polymer solution that underwent crosslinking by the extract concentration in the same solution (1%, 2.5%, or 5%) and the glucoraphanin fraction of the *Brassica oleracea* extract (22.10%), as obtained by HPLC-DAD measurements); and *GRF_lost_* is the total amount of glucoraphanin lost in the crosslinker and rinsing solutions, determined via HPLC-DAD analyses, as reported in Section 3.3.

In this case, the EE% was not 100% because some of the glucoraphanin originally present in the polymeric solution used to synthesize the hydrogel beads was lost in the crosslinking saline solution during the gelation process. To determine the amount of glucoraphanin lost during this step and during the subsequent rinsing steps, both the crosslinker solution and the water used to rinse the hydrogels were collected and analyzed by means of HPLC-DAD, as described in Section 3.3, using an external calibration in the linear concentration range of 0.1–20 ppm.

#### 3.8.2. Release Kinetics in Water and Release Efficiency (RE%)

To study the release kinetics in water and the release efficiency (RE%), a known amount of the loaded hydrogels was immersed in water (hydrogel/water 1:10, *v*/*v*) and kept under magnetic stirring to favor the release of glucosinolates. Also in this case, the analysis focused on the release profile of glucoraphanin as a representative of the glucosinolates loaded into the hydrogel. Several 100 μL samples were taken over three hours of observation, each time replacing the sampled volume with fresh water to maintain a constant volume. The samples were then diluted 1:100 and analyzed by means of HPLC-DAD, as described in Section 3.3, using an external calibration in the linear concentration range of 0.1–20 ppm. All measurements were performed in triplicate.

## 4. Conclusions

The potential of Ca^2+^/alginate (ALG)- and Fe^3+^/carboxymethyl cellulose (CMC)-based hydrogel beads as vehicles for the controlled release of glucosinolates in agricultural soils was explored, with the aim of searching for improved and innovative treatments inspired by biofumigation. A commercial *Brassica oleracea* extract was first characterized through HPLC-MS-DAD analyses, and it was shown that the total glucosinolate content was around 28% *m*/*m*, with glucoraphanin being the most abundant, while 12% *m*/*m* of sinapine was also detected, which can contribute to the biocidal properties of the product. The extract was then loaded (exploring a 1–5% concentration range, representing the initial extract concentration in the pre-crosslinking polymeric solution) into hydrogel beads, which were physico-chemically characterized through DSC, rheometry, and SEM, while the encapsulation efficiency (EE%) and release efficiency (RE%) in water were investigated through HPLC-DAD analyses. The ALG and CMC hydrogels were shown to share an equilibrium water content (EWC) of 93% and a high free water index (FWI), which showed opposite trends in the presence of increasing extract concentration for the two polymeric networks. While the FWI increased for the ALG systems, indicating that glucosinolates likely replaced water molecules in the interaction with alginate chains, it dropped for the CMC systems, possibly due to the formation of hydrated Fe^3+^/glucosinolate complexes. By looking at G’ and G’’, the rheological measurements confirmed the hypothesis, showing that the CMC hydrogels possessed higher mechanical properties than the ALG ones. A slight difference between the two polymer networks was also observed with respect to the EE%, which was higher for the CMC-based hydrogels. This could be due to a combination of factors, including the inner structure and the surface-to-volume (S/V) ratio of the beads. Indeed, the SEM micrographs of the CMC hydrogels showed thicker and more robust gel walls, which could better confine the active compounds inside the beads. On the other hand, the lower viscosity of the alginate polymer solution was reflected in smaller hydrogel beads, which had a higher S/V ratio, accounting for favored glucosinolate migration from the gel to the surrounding aqueous environment. The RE% (measured in water) was higher for the ALG hydrogels, even though the release profiles, fitted using the Weibull model function, showed a gradual and slow release for both systems, which tended to reach an asymptotic value after almost 3 h of hydrogel immersion in water. This would enable a prolonged and controlled effect in agricultural soils.

Overall, both proposed systems possess excellent properties for real *in-field* applications. However, the CMC-based hydrogels seem to be preferable due to their slightly higher EE%, better mechanical properties, and better retention of extract active molecules, which could result in a more gradual and prolonged effect during soil treatment. For these reasons, and possibly due to the enhanced contributions of Fe(III) to the biocidal properties of gel beads, the CMC-based system seems slightly more promising for further testing.

Further experiments will include the investigation of release profiles in artificial model soils and—most importantly—in real agricultural soils, even though the organic compounds naturally present in soils complicate the analysis due to possible interference with the analytes’ quantification.

This work clearly demonstrated the potential of cheap, simple, and eco-friendly hydrogels as carriers for active compounds, possibly extracted from plants or byproducts of agrifood production, as an advanced tool for agricultural soil treatment. The versatility of these materials enables their use in other applicative fields, such as the cleaning of biodegraded artistic surfaces in the context of cultural heritage conservation, among others.

## Figures and Tables

**Table 1:** Retention times and fragment ions for the identification of the components in the *Brassica oleracea* extract.

Analyte	Retention Time (min)	[M − H]^−^	Fragment Ions
Glucoraphanin	1.91	436	292, 275, 259, 194, 130
4-Hydroxyglucobrassicin	4.4	463	383, 285, 275, 267, 259, 240
Glucoerucin	8.93	420	340, 291, 275, 259, 242, 227, 224, 195, 178
Glucobrassicin	9.55	447	275, 259, 251, 205
Sinapine	14.09	354	294, 279, 264, 223, 208

**Table 2:** FWI measured in ALG-based and CMC-based hydrogels in the 0–5% concentration range.

Sample	ΔH_exp_ (J/g)	FWI (%)
ALG	238 ± 12	77 ± 4
ALG-1	242 ± 12	78 ± 4
ALG-2-5	279 ± 14	90 ± 5
ALG-5	288 ± 14	93 ± 5
CMC	265 ± 13	86 ± 4
CMC-1	217 ± 11	70 ± 4
CMC-2.5	179 ± 9	58 ± 3
CMC-5	165 ± 8	53 ± 3

**Table 3:** Weibull model fitting parameters for the release profiles in water for all investigated hydrogels.

System	*K*	*a*	*b*	Max Glucoraphanin Released ^a^
CMC 1%	62%	0.16	0.48	0.6 ± 0.1
CMC 2.5%	39%	0.46	0.41	1.4 ± 1.0
CMC 5%	35%	0.38	0.42	3.2 ± 1.0
ALG 1%	73%	0.50	0.42	0.7 ± 0.1
ALG 2.5%	46%	0.53	0.41	1.5 ± 1.0
ALG 5%	44%	0.42	0.38	3.3 ± 0.2

^a^ The concentration (ppm) of glucoraphanin, measured in the solution in which the hydrogels were immersed, after 3 h of release.

## Data Availability

Data are contained within the article.
